# Knowledge and practices of malaria prevention with ITNs in post-and near-elimination areas of Vanuatu

**DOI:** 10.1186/1475-2875-11-S1-P59

**Published:** 2012-10-15

**Authors:** Peter S Larson, Akira Kaneko, Koji Lum, Noriko Watanabe, Takeo Tanihata

**Affiliations:** 1 University of Michigan School of Public Health, Ann Arbor, Ml, USA; 2 Osaka City University, Osaka, Japan, Karolinska University, Stockholm, Sweden; 3 State University of New York, Binghamton, New York, USA; 4 Osaka City University, Osaka, Japan; 5 Ministry of Science and Technology, Tokyo, Japan

## Background

Insecticide Treated Nets (ITNs) remain an important tool for sustained malaria control and play an integral part in malaria elimination strategies. As malaria incidence decreases in holodemic areas, however, proactive and regular use of ITNs may simultaneously decline if risk perception diminishes.

## Data/methods

In Summer 2012, we conducted a cross-sectional survey of three communities in Vanuatu: i) where malaria has been locally eliminated (Aneityum), ii) where malaria remains present but with rapidly declining incidence (Ambae), and iii) an urban area where malaria transmission may or may not occur (Efate). Respondents were asked a battery of questions regarding knowledge of malaria, ITN possession and use, and compliance with other anti-malaria interventions. Information on basic demographics, education levels, dietary habits and household economic activities were also recorded.

## Results

Residents of Aneityum (malaria eliminated) reported near universal use of ITNs, but uneven knowledge of malaria, particularly in younger individuals born around the time of malaria elimination. Residents in the other communities reported less consistent, though high levels of ITN use despite past individual malaria diagnoses.

## Conclusions

Results indicate that achieving sustained high levels of ITN use in near- and post-elimination contexts is possible, but that maintaining awareness could present a long-term challenge to prevent reintroduction and recrudensence. Sustained local community cooperation will be essential to maintaining elimination efforts worldwide.

